# Influence of Hydrocolloids on Lipid Digestion and Vitamin D Bioaccessibility of Emulsion-Filled Soft Gels

**DOI:** 10.3390/gels11120964

**Published:** 2025-11-29

**Authors:** Carla Arancibia, Cristóbal Rojas, Matías Meneses, Karen Vielma, Teresa Vásquez, Natalia Riquelme

**Affiliations:** Laboratorio de Investigación en Propiedades de los Alimentos (INPROAL), Departamento de Ciencia y Tecnología de los Alimentos, Facultad Tecnológica, Universidad de Santiago de Chile, Obispo Umaña 050, Estación Central-Santiago 9170201, Chile

**Keywords:** older people, emulsion-based gels, hydrocolloids, physical properties, lipid digestion, vitamin D bioaccessibility

## Abstract

The global increase in the older population presents a nutritional challenge; therefore, the development of food products for this group must take into account the physiological changes associated with aging. This work aimed to evaluate the effects of droplet size, conventional emulsion (CE) and nanoemulsion (NE), and different hydrocolloids, soy protein (SPI), whey protein (WPI), agar (AG), and κ-carrageenan (CAR), on the physical properties, lipid digestibility, and bioaccessibility of emulsion-based gels enriched with vitamin D. The main findings indicated that all gels exhibited non-Newtonian behavior and suitable viscosity and texture for the swallowing needs of older people. The highest release of free fatty acids (~30%) was observed in the NE + WPI sample, independent of droplet size. Instead, SPI gels showed the highest vitamin D bioaccessibility, likely due to their less-structured gel network. Thus, gels containing WPI + AG provide a favorable balance between an easy-to-swallow texture and efficient nutrient release, making them suitable for producing food based on emulsion-filled gels with good physical and nutritional properties. Hence, these results highlight the potential of tailored hydrocolloid combinations to develop nutrient-fortified and texture-appropriate gels that address the nutritional needs of the older population.

## 1. Introduction

The World Health Organization projects that the global population aged 60 years and older will double over the coming decades, reaching 2.1 billion by 2050 [1]. These demographic changes highlight the need to address the challenges associated with aging, as older adults experience various physiological changes. For instance, aging often leads to conditions like dysphagia, which impairs swallowing efficiency and increases the risk of aspiration pneumonia [2]. Additionally, gastrointestinal disorders, such as reduced secretion of digestive fluids, weakened peristaltic contractions, and suboptimal gastric emptying, can compromise nutrient bioaccessibility by limiting enzymatic hydrolysis and nutrient absorption [3,4]. Moreover, older adults frequently consume below the recommended levels of macronutrients (e.g., proteins, fiber, and essential fatty acids) and micronutrients (e.g., vitamins B_6_, B_12_, D, E, and K) [5,6]. As a result, food fortification for this population is essential to prevent age-related conditions and enhance overall health and well-being [7,8]. In particular, vitamin D is a steroid hormone that contributes to human well-being by promoting a strong skeletal system, facilitating muscle movement, supporting the immune system, and optimizing cellular function [9]. In older adults, vitamin D deficiency is associated with an increased risk of autoimmune, cardiovascular, and infectious diseases, as well as the development of certain cancers [10]. However, functional foods designed with modified textures that promote safe swallowing and enhance nutrient bioaccessibility present both nutritional and technological challenges, emphasizing the need to develop food structures specifically tailored to the needs of older adults.

Emulsion-filled gels are semi-solid systems characterized by a gel network structure that typically contains embedded oil droplets [11]. Due to their unique properties, these gels show strong potential as texture-modified foods. They can improve mouth feel and lubrication, which is especially beneficial for individuals with swallowing difficulties [12]. Moreover, they can act as effective carriers for lipophilic bioactive compounds, protecting them during gastrointestinal transit and enhancing both their bioaccessibility and bioavailability [13,14]. Although emulsion-filled gels have been used to reduce the fat content of food products, few studies have investigated their potential to enhance the stability and absorption of lipid-based nutrients [15,16,17,18]. Therefore, the use of these structures as delivery systems that enable site-specific release in the gastrointestinal tract and provide tailored textural characteristics represents a novel solution to meet the needs of older adults.

Hydrocolloids, which include both proteins and polysaccharides, can serve as effective structuring materials for designing emulsion-filled gels [11,19,20]. Despite their potential, few studies have focused on using these systems to develop food products specifically for older adults. For example, Wang et al. [21] investigated the effect of whey protein morphology (fibrous and granular structures) on the texture properties of emulsion gels. They assessed its potential for creating soft-textured, homogeneous foods suitable for seniors. Similarly, another study explored the development of nanoemulsion-based gels using various gelling agents (agar and carrageenan) to meet the textural needs of older adults, examining the physical properties of these gel systems [22]. It is important to note that these studies did not consider hydrocolloid mixtures. However, the evidence indicates that the type and concentration of hydrocolloids could significantly impact the physical properties of emulsion gel systems. In this regard, proteins and polysaccharides can synergistically form mixed emulsion-filled gels with different gel structures [23,24,25], since the combination of protein isolates with agar or carrageenan contributes to network formation and interfacial stabilization, while agar and carrageenan provide complementary gelling mechanisms to those of proteins [26]. Additionally, older adults have a high protein requirement, around 1–1.6 g/kg/day [27]. Therefore, the use of two types of hydrocolloids can be a helpful strategy to improve the nutritional profile of these food structures, thereby enhancing food diversity and ensuring adequate intake of this essential macronutrient for this population group. This approach presents a promising strategy for developing functional emulsion-filled gel foods specifically for the older population, addressing both their nutritional and textural needs [28].

Regarding proteins, whey proteins are widely used in food products due to their high nutritional value, balanced amino acid profile, health benefits, and functional properties [29]. However, plant proteins have become more popular due to consumers’ ethical and environmental concerns. Soy protein is advantageous in plant-based foods; it offers a good amino acid profile, useful functional properties (e.g., gelling), and is low cost [30]. On the other hand, polysaccharides are widely studied for their versatility and benefits to gut microbiota [31]. Agar and κ-carrageenan, red algae-derived polysaccharides, are commonly used as gelling agents. Although, their gelation differs, agar forms gels via a thermal sol–gel transition with double helix aggregation on cooling, while κ-carrageenan requires specific cations (K^+^, Ca^2+^) for helix formation [32]. Therefore, their use in combination with proteins could allow modulation of gel structure and the design of foods tailored to the needs of the elderly.

In this context, this study makes a distinct contribution to the field by being among the first to enhance the bioaccessibility of vitamin D encapsulated in emulsion-filled gels for older adults, utilizing innovative hydrocolloid mixtures. Specifically, it uniquely investigates the combined use of whey and soy protein isolates with polysaccharides, such as agar and κ-carrageenan. It evaluates the effect of micro- to nanoscale emulsion droplet sizes. By examining how these variables interact, this research provides new and original insights into the design of emulsion-filled gels as targeted delivery systems for lipid-based micronutrients in age-friendly food applications.

## 2. Results and Discussion

### 2.1. Emulsion Characterization

Table 1 presents the physical characteristics of the emulsions used to prepare the emulsion-filled gels. As expected, varying high-pressure homogenization conditions resulted in emulsions with micro- and nano-scale droplet sizes: 20.7 µm for conventional emulsion (CE) and 194 nm for the nanoemulsion (NE). According to Mushtaq et al. [33], the formation of nanoemulsions typically requires significant energy to create tiny oil droplets, and droplet size is directly related to the energy applied during homogenization [34]. Therefore, the NE was prepared under more extreme homogenization conditions (700 bar and 5 cycles) than the CE (300 bar and 1 cycle), resulting in smaller droplet sizes in the NE system.

**Figure 1:**
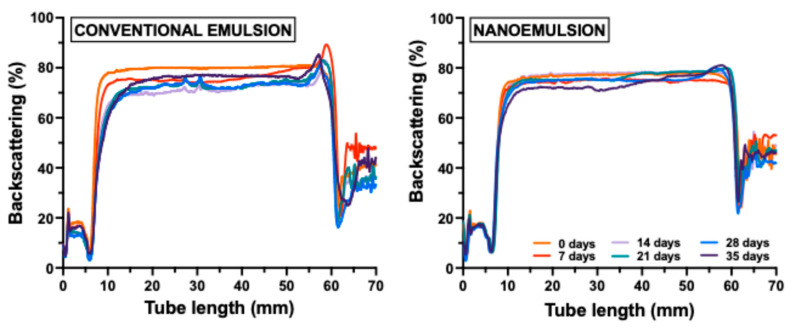
Backscattering profile of the emulsions with different droplet sizes during 35 days of storage at 5 °C.

Regarding the physical stability of emulsions, Figure 1 illustrates the backscattering (BS) profiles of CE and NE over 35 days of storage at 25 °C. Different behaviors were observed between emulsion types. The midpoint BS percentage (measured between 10 and 50 mm) decreased gradually over time during storage in both emulsions. The decrease was more pronounced in CE (from 7 days) compared to NE (from 21 days) (Figure 1). This behavior suggests that the emulsion destabilization occurs through a flocculation and/or coalescence mechanism [35]. Besides, these differences (a creaming index of 9.4% at the end of storage time for CE versus 3.8% for NE) can be attributed to differences in oil droplet size. McClements [36] mentioned that emulsion stability can be enhanced by reducing oil droplet size. Conversely, emulsions with smaller oil droplets (such as NE) demonstrate greater stability. Finally, this improved stability can also be attributed to interfacial properties, such as repulsive forces, which reduce flocculation and coalescence [37]. In this sense, when the droplets are smaller, the total surface area of the system increases, allowing more complete coverage of the interface by ionic emulsifiers (such as soy lecithin). This promotes electrostatic repulsion and, consequently, enhances emulsion stability.

Additionally, this phenomenon of emulsion destabilization may lead to phase separation [38], as illustrated in Figure 1. The BS (%) in the upper part of the tube (50–60 mm) increased over storage time. This result indicates the formation of a creaming layer in the CE. This result corroborates the lower physical stability due to the larger droplet size, as previously mentioned. Likewise, significant differences (*p* < 0.05) in creaming index values were observed between emulsions (Table 1). The CE showed a higher creaming index than the NE. The CI values after 35 days of storage at 5 °C were 9.4 and 3.8% for NE and CE, respectively.

### 2.2. Characterization of Emulsion-Filled Gels

**Figure 2:**
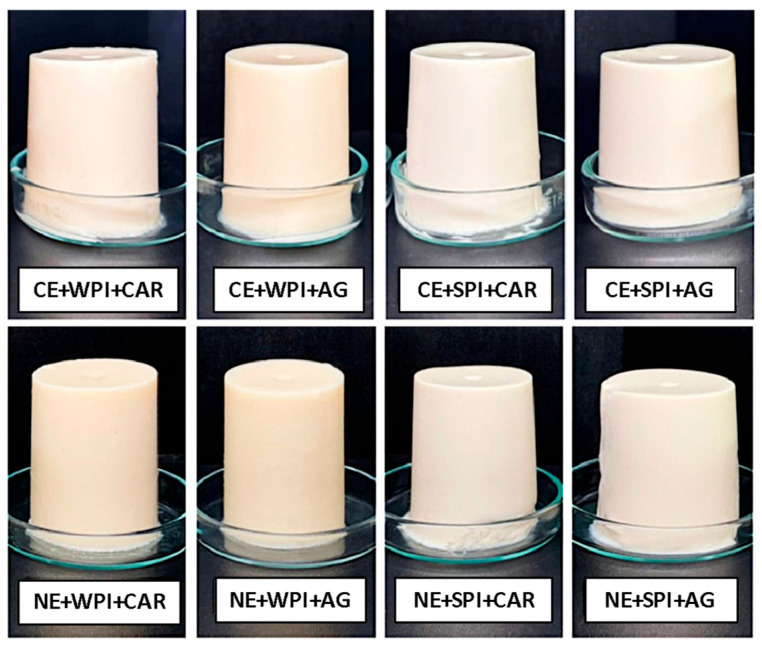
Photographs of emulsion-filled gels with different droplet sizes and hydrocolloid type. CE: conventional emulsion, NE: nanoemulsion, WPI: whey protein isolate, SPI: soy protein isolate, CAR: κ-carrageenan, and AG: agar.

According to Farjami & Madadlou [39], the emulsion gels obtained in this study are classified as emulsion-filled gels since the oil phase fraction (10%) was too low to create a network of flocculated droplets. Therefore, the formation of the emulsion-based gels depended mainly on heat-induced gelation. The images of emulsion-filled gels are displayed in Figure 2.

As shown, all samples were self-supporting, with good integrity, and exhibited a white, opaque appearance, characteristic of the emulsion composition. This appearance results from light-scattering by oil droplets [40]. While these similarities in appearance are notable, the type and nature of the biopolymers remain important factors in studying the interactions and structures of protein–polysaccharide gels [41]. Therefore, the rheological and textural properties of emulsion-based gels were examined to compare their internal structure based on the use of different hydrocolloid mixtures.

#### 2.2.1. Flow Behavior

**Figure 3:**
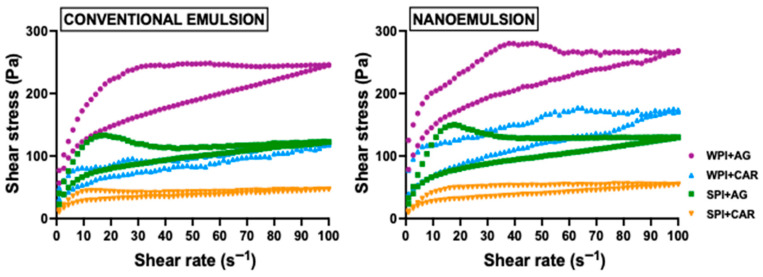
Flow curves of the different emulsion-filled gels with different droplet sizes and hydrocolloid type. WPI: whey protein isolate, SPI: soy protein isolate, CAR: κ-carrageenan, and AG: agar.

Figure 3 shows the flow curves of emulsion-filled gels. All samples exhibited non-Newtonian and pseudoplastic behavior. The shear stress of the samples decreased with increasing shear rate. These results indicate shear-thinning properties, which can be beneficial for safe swallowing. At low shear speeds, when food is on the tongue, gels behave like a thick fluid, and as shear increases during swallowing, a fluidification effect occurs. This behavior helps manage the passage of the gel through the oral cavity and slows flow in the pharyngeal region [42]. These findings suggest that emulsion-filled gels may be suitable for incorporation into foods formulated for older people with swallowing disorders, since their flow behavior helps reduce the risks of aspiration and food retention.

In addition, the gels exhibited thixotropic behavior, as shown by the hysteresis area between upward and downward curves (Figure 3). This effect was more pronounced in agar-based gels. This behavior occurs due to structural collapse during shearing, in which the structure fails to fully recover after shear [43]. The pronounced hysteresis area in AG-based gels suggests they break more easily during shearing [44]. This result can be an advantage in creating a food bolus that slides more easily into the oral cavity.

To determine the flow parameters, the downward flow curve data were fitted to the Ostwald-de Waele model (R^2^ > 0.96). The consistency index, flow index, and apparent viscosity were determined at a shear rate of 50 s^−1^. These values are presented in Table 2. Both protein and polysaccharide types have a significant effect (*p* < 0.05) on the consistency index (*K*). Gels made with whey protein (WPI) showed higher *K* values than those with soy protein (SPI) (Table 2). This difference likely relates to structural and molecular differences in the gels formed by each protein, in which the thermal process can alter protein secondary structure and reorganize their interactions. In this case, we can hypothesize that WPI unfolds during thermal processing to form larger spherical aggregates, thereby enhancing protein–protein interactions and promoting the formation of structured, firm gels [45]. In contrast, heated SPI forms weak non-covalent interactions among smaller protein aggregates, resulting in softer and less consistent gels [30]. On the other hand, gels made with agar (AG) also showed significant differences in *K* values (*p* < 0.05) compared to κ-carrageenan (CAR) ones, particularly in WPI-based gels (Table 2). When AG is heated and cooled, it can form rigid, firm gels due to its molecular structure, in which the double helix configuration of its chains (β-1,3-bound D-galactose and α-1,4-bound 3,6-anhydrous L-galactose) creates a strong network [46], potentially exerting a synergistic effect with WPI. Similar results were reported by Ding et al. [47], who developed zein–alginate nanogels in which zein and alginate form a tightly bound structure via electrostatic adsorption, hydrophobic interactions, and hydrogen bonding. In contrast, CAR contains fewer helical regions, leading to reduced aggregation among its polymer chains and, consequently, to less uniform and weaker gels [48]. Regarding particle size, the interactions between AG and WPI induce significant changes in the *K* values of nanoemulsion-based gels compared to conventional emulsion-based ones (Table 2). This effect may be attributed to the synergistic interaction between these hydrocolloids, which promotes an even distribution of small droplets throughout the gel matrix, thereby enhancing its consistency.

In addition, all samples exhibited flow index (*n*) values less than 1, supporting their pseudoplastic behavior (Table 2). Only the interaction between protein and polysaccharide type significantly affected the flow index (*p* < 0.05). For SPI samples, CAR-based gels showed significantly lower *n* values (*p* < 0.05) than AG-based ones. Conversely, WPI samples showed the opposite effect: AG-based gels were more pseudoplastic than CAR-based samples (Table 2). These findings suggest that the protein–polysaccharide interactions in these gels are primarily physical, such as electrostatic, hydrophobic, Van der Waals forces, and hydrogen bonds [28]. For example, CAR interacted with the hydrophilic groups of SPI through electrostatic interactions and hydrogen bonding [49]. Similarly, WPI form hydrophobic and hydrogen-bond interactions with AG [50]. These interactions allow for better molecular alignment during shear flow and, therefore, decrease their pseudoplasticity.

Apparent viscosity was measured at a shear rate of 50 s^−1^ (η_50s−1_), which relates to the shear experienced in the mouth when consuming semi-solid food [51]. This parameter may be relevant for foods intended to dysphagic patients, since a higher bolus viscosity enhances bolus control and swallowing safety in older adults [52]. ANOVA results indicated that protein and polysaccharide type significantly affected η_50s−1_ values (*p* < 0.05). Like the consistency index, WPI + AG samples had greater viscosity (*p* < 0.05) than other samples (Table 2), while SPI + CAR samples showed the lowest η_50s−1_ values (924 and 925 Pa s for CE and NE, respectively). These differences may result from interactions between proteins and polysaccharides during gelation, specifically the negatively charged sulfate groups in agar’s polymer chains, which cause polysaccharide-protein complexes [15]. These complexes can contribute to a more structured gel with greater resistance to flow. Despite these differences, most tested gels had η_50s−1_ greater than 1000 Pa s. According to the International Dysphagia Diet Standardization Initiative (IDDSI), gels are categorized as thick foods (level 4) because they do not require mastication, which benefits older adults by making the bolus easier to manage during consumption [53]. The flow behavior of foods for older adults is especially relevant during swallowing, particularly in the pharyngeal phase [54]. Therefore, these findings suggest improved swallowing safety as part of dysphagia management in older people.

#### 2.2.2. Texture Properties

**Figure 4:**
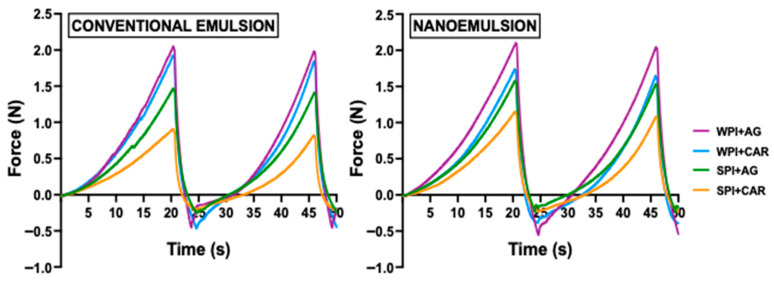
Texture profile curves of emulsion-filled gels with different droplet sizes and hydrocolloid type. WPI: whey protein isolate, SPI: soy protein isolate, CAR: κ-carrageenan, and AG: agar.

A texture profile analysis (TPA) characterized the mechanical behavior of the samples. This test simulates chewing with two compressions, representing the first and second bites [55]. Figure 4 presents force-deformation versus time curves for the gels, which show distinct texture profiles among samples. Nanoemulsion (NE)-based gels had slight differences in the maximum force-deformation peak (the highest force during compression) compared to conventional emulsion (CE) gels. WPI gels required a higher compressive force than SPI gels. The polysaccharide type also affected the compression force: AG-based gels exhibited higher force-deformation peaks than CAR-based gels. This pattern matches the flow property results (Figure 3 and Table 2). Therefore, we hypothesize that WPI samples can form covalent bonds with polysaccharides (AG and CAR), thus exerting the strongest intermolecular forces. Instead, SPI gels exhibit weaker intermolecular interactions due to their compact, aggregated structure [56].

From the TPA curves, we obtained the hardness, adhesiveness, and cohesivity parameters (Table 3). Ibañez et al. [57] found that these textural parameters can influence the swallowing reflex and the ease with which people swallow food. Thus, these parameters are essential considerations when developing food products for older adults. In this way, the ANOVA results showed that protein and polysaccharide types significantly affected (*p* < 0.05) the hardness, adhesiveness, and cohesiveness values of emulsion-filled gels. Gels elaborated with WPI showed higher hardness (495–682 N/m^2^) than those with SPI (297–412 N/m^2^). AG-based gels also had higher hardness (353–682 N/m^2^) than CAR-based gels (297–509 N/m^2^) (Table 3). No differences in the hardness of gels with different droplet sizes were found, except between NE + SPI + AG gels and CE + SPI + AG, which show a decrease in hardness with increasing droplet size of emulsions (Table 3). The IDDSI states that hardness values below 1500 N/m^2^ indicate textures that do not require chewing [58]. In these foods, tongue compression is sufficient to break them [59], making swallowing easier. Therefore, all gels studied are suitable for older people’s diets, despite varying hardness values.

Adhesiveness is the effort required to overcome the attractive forces between food and the tongue or palate, where lower adhesiveness values mean it is easier for older adults to swallow [60]. Samples containing SPI were less adhesive (0.02–0.04 J/m^2^) than gels with WPI (0.05–0.06 J/m^2^) (Table 3). The type of polysaccharide had a minimal effect, although significant differences were seen between the CE + SPI + CAR and NE + WPI + AG samples. Despite these differences, all emulsion-based gels remain suitable for older people. The Japanese Society of Dysphagia Rehabilitation states that foods with adhesive values greater than 1000 J/m^2^ are a risk to people with swallowing problems [61], as the sticky nature of such foods may increase the risk of pharyngeal waste [57,62]. The low adhesiveness of the gels obtained (<0.06 J/m^2^) suggests favorable textural properties for bolus formation and oral transport, which are desirable characteristics for foods designed to assist in dysphagia management among older adults.

The cohesiveness parameter shows the sample’s internal structure and relates to the fluidity of the food bolus during oral processing and swallowing [54]. As noted, cohesiveness values differed significantly (*p* < 0.05) based on the type of protein and polysaccharide. Gels with whey protein and agar (WPI + AG) had the highest cohesiveness (~0.6), regardless of emulsion type (CE or NE). This result likely stems from hydrophobic interactions between the hydrocolloids, which increase cohesiveness. In agar-based emulsion-gel systems, oil droplets are distributed uniformly within the polysaccharide network [15], thereby enhancing cohesiveness. In contrast, CAR-based gels were less cohesive because κ-carrageenan molecules aggregated and reticulated around the oil droplets [63]. Cohesiveness values for texture-modified foods for individuals with dysphagia typically range from 0.2 to 0.9 [61]. Thus, all emulsion-based gels exhibit internal structural cohesion, which helps prevent uncontrolled fragmentation into small particles during swallowing [20]. This finding is consistent with the flow behavior analysis, which shows that the shear-thinning behavior of the gels allows them to deform and flow under oral forces without fragmenting, making them suitable for older adults.

Finally, all emulsion-based gels developed in this study are appropriate for the older population with swallowing difficulties, as they exhibit appropriate cohesiveness, lower hardness and adhesiveness, and require minimal chewing effort for consumption.

### 2.3. In Vitro Digestion of Emulsion-Based Gels

Because emulsion-based gels can serve as delivery vehicles for lipid bioactive compounds, it is crucial to understand their behavior during in vitro digestion, particularly under the physiological conditions of older adults.

#### 2.3.1. Lipid Digestion

**Figure 5:**
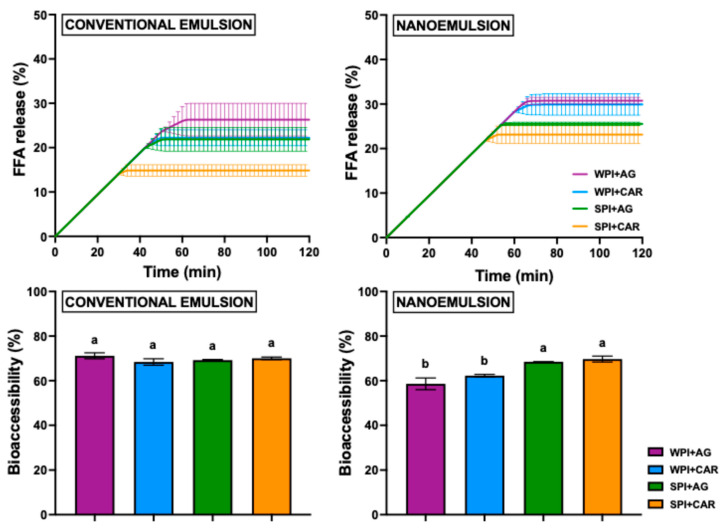
Lipid digestion and vitamin D bioaccessibility of emulsion-filled gels with different droplet sizes and hydrocolloid type. WPI: whey protein isolate, SPI: soy protein isolate, CAR: κ-carrageenan, and AG: agar. Different letters indicate significant differences (Fisher; *p* < 0.05) in the bioaccessibility for different samples.

The release profile of free fatty acid (FFA) during in vitro intestinal digestion is shown in Figure 5. The percentage of FFA rose linearly in the initial minutes, demonstrating first-order kinetics. This %FFA increase generally occurred within the first 50 min of digestion. In contrast, the gel CE + SPI + CAR exhibited a rise in %FFA as early as 30 min (Figure 5). During this period, faster fatty acid release reflects lipid hydrolysis as lipase interacts with the surface of emulsified oil droplets [64]. Then, it is expected that lipid products can incorporate into mixed micelles, clearing the oil droplet surfaces for ongoing lipase activity [65], as evidenced by the later digestion time, where the top of lipid hydrolysis occurs. Subsequently, the plateau of %FFA curves indicated that no further fatty acid release occurred by the end of intestinal digestion [66]. This phenomenon is likely due to the surface activity of digestion products (FFAs, mono and diglycerides), which may compete with lipases for adsorption at the oil–water interface, thus restricting triglyceride hydrolysis [64,67,68].

On the other hand, the total %FFA released by the end of the intestinal phase was used to compare the digestion of samples. Figure 5 shows lower free fatty acid release after intestinal digestion, with values ranging from 14.9 to 30.8%. This low lipid digestibility may be due to gastrointestinal conditions in older adults in the in vitro assays. Older populations exhibit significantly reduced proteolysis and lipolysis during digestion, resulting in decreased nutrient digestibility [69]. Additionally, low FFA release may occur due to the formation of aggregates or undigested proteins, particularly in plant-based ones, which create a physical barrier that hinders lipase-oil droplet contact during intestinal digestion [19]. Due to sample differences, a three-way ANOVA was performed, showing that protein, polysaccharide, and emulsion types significantly affected lipid digestion (*p* < 0.05) in emulsion-based gels. First, the polysaccharide type has not significant effect (*p* > 0.05) on lipolysis. Gels containing CAR released fewer FFAs compared to those with AG (Figure 5). This slight variation may be due to CAR gels having a weaker structure, making the matrix more susceptible to rupture and oil droplet release during the gastric phase, potentially leading to droplet displacement and increased coalescence [70].

Regarding droplet size, NE-based gels showed a significantly higher %FFA released than CE-based gels (by 6%), regardless of the protein or polysaccharide used (Figure 5). These differences may be due to the smaller droplet size of nanoemulsified gels, which showed a higher surface area for lipase adsorption on oil droplets, promoting fatty acid release [71]. Furthermore, Das et al. [72] indicated that systems with larger oil droplets (conventional emulsions) are more susceptible to flocculation during the gastric phase. This inhibits lipase access to the droplet surface during the intestinal phase [73], resulting in decreased lipid digestion.

In the case of protein type, a significant difference between proteins was observed. WPI-based gels released more fatty acids (~30% FFAs) than SPI gels (~20% FFAs) (Figure 5). This difference may result from the greater solubility and interfacial capability of whey proteins. The gastric environment (pepsin, acidic pH, and mechanical agitation) disrupts the gel matrix and releases oil droplets, inducing emulsion destabilization by coalescence [74]. Here, WPI’s superior interfacial activity enables efficient adsorption at the interface, lowering the interfacial tension of oil droplets and delaying coalescence during the gastric phase, thereby enhancing intestinal lipid digestion [71]. In contrast, SPI has poor emulsifying properties due to its complex, compact, and aggregated structure. Soy protein contains numerous hydrophobic groups, which impair its solubility and emulsification, and promote oil droplet coalescence during digestion [75]. This limited emulsifying efficiency of soy protein hindered fatty acid release during the intestinal phase.

With respect to gel structure, lipid digestion behavior was also influenced by the structural properties of the gels. Gels formulated with WPI, particularly those combined with AG, exhibited a more compact network (Figure 3 and Figure 4, Table 2 and Table 3); however, this structure was more susceptible to proteolysis, resulting in a higher FFA release (Figure 5). In contrast, SPI-based gels formed less compact networks with homogeneously entrapped lipid droplets, which limited lipase accessibility and therefore led to lower FFA release due to reduced soy protein degradation. Overall, we hypothesize that gel structure and the extent of proteolysis affect the lipid digestibility of emulsion-based systems, indicating that protein source selection is a key factor in modulating lipolysis in these matrices.

Finally, an optimized combination of protein, polysaccharide, and emulsion type (defined by their droplet size), such as gels with NE + WPI + AG (with the highest %FFA), can promote lipid digestibility. This improvement is due to the stability of oil droplets during the gastric phase, where fewer droplets were released from the gel network due to its highly cross-linked structure [76]. Consequently, oil droplets retain their size throughout the gastric phase, promoting FFA release in the intestinal phase [77]. Therefore, this gel formulation (NE + WPI + AG) offers potential to improve the bioaccessibility of various lipid bioactive compounds incorporated into nanoemulsions.

#### 2.3.2. Vitamin D Bioaccessibility

Figure 5 shows the vitamin D bioaccessibility from emulsion-filled gels after in vitro digestion, which slightly varied between 58 and 71%. Regarding protein type, no differences in vitamin D bioavailability were observed between NE and CE gels based on SPI (Figure 5), since all formulations exhibited similar vitamin D bioaccessibility values (68.48–70.02%). Instead, WPI-based gels showed significant differences (*p* < 0.05) by droplet size. NE + WPI gels obtained lower vitamin D bioaccessibility than CE + WPI, regardless of polysaccharide type. In addition, it was expected that the samples showing the highest free fatty acid release would also exhibit the greatest vitamin D bioaccessibility (Figure 5). However, the observed results did not follow this pattern. The NE + WPI + AG and NE + WPI + CAR samples, which have the highest percentage of free fatty acids (FFAs) released (Figure 5), showed the lowest vitamin bioaccessibility (58.6% and 62.3%, respectively). These results suggest a limited protective effect of WPI gels on vitamin D. It should be noted that vitamin bioaccessibility depends on the release of vitamins from oil droplets embedded within the protein matrix, followed by their solubilization into mixed micelles [78]. Thus, we hypothesize that WPI undergoes a higher degree of proteolysis, resulting in reduced vitamin D bioaccessibility. This could be attributed to the fact that WPI hydrolysis in the gastric environment promotes the release of vitamin D from the protein matrix, which in turn increases its susceptibility to oxidative degradation or to interactions with newly generated hydrophobic peptide fragments, ultimately limiting its bioaccessibility [79,80].

#### 2.3.3. Microstructure

**Figure 6:**
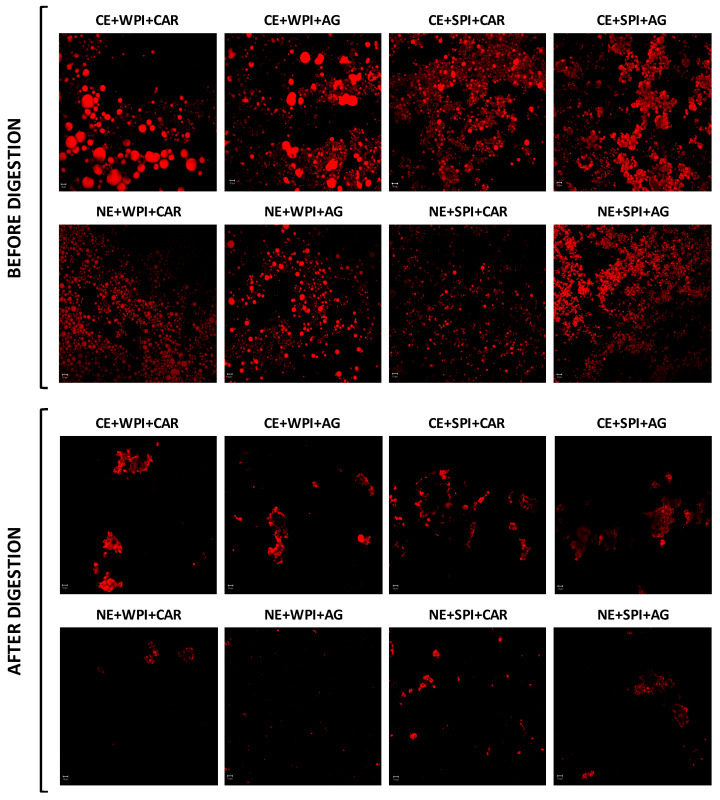
Confocal images of the gels before and after in vitro digestion. CE: conventional emulsion, NE: nanoemulsion, WPI: whey protein isolate, SPI: soy protein isolate, CAR: κ-carrageenan, and AG: agar. Scale bars represent 10 μm in length, and red areas represent lipid regions.

Confocal laser microscopy (CLSM) was used to investigate the changes in the microstructure of emulsion-based gels during in vitro digestion (Figure 6). Before digestion, CLSM images revealed that SPI samples had a denser network than WPI samples, probably due to larger agglomerates formed by the more compact structure of soy proteins [81]. Additionally, WPI gels exhibited a higher number of flocculated and coalesced oil droplets, whilst SPI gels showed the highest number of encapsulated oil droplets between protein chains, controlling destabilization of the oil droplets (Figure 6). This difference may be attributed to protein–protein and protein–oil–water interface interactions. SPI exhibits more hydrophobic regions, which promote its adsorption at the oil–water interface and, consequently, oil droplet stability [82]. Instead, WPI readily unfolds upon heating (during gel formation), which favors protein–protein interactions and destabilizes oil droplets [74]. These results are consistent with the textural properties of the gels, in which SPI gels form a weaker network than WPI gels (Section 4.2). Regarding emulsion type, a more uniform distribution of oil droplets within the gel matrix was observed in NE-based gels, whilst CE samples showed pronounced flocculation and coalescence of oil droplets due to the larger droplet size and the heat treatment used during gel preparation.

After in vitro digestion, structural changes were detected in the samples. According to Lu et al. [83], gel networks collapsed due to the action of gastrointestinal enzymes, shearing, pH shifts, and changes in ionic strength. In particular, the structural breakdown of protein chains facilitated the release of oil droplets from the gel, promoting the adsorption of lipase onto oil droplets and, consequently, their digestion. In this sense, gels with SPI showed a greater presence of protein aggregates with lipid residues after digestion compared to WPI gels (Figure 6), especially in NE-based gels. This result can be related to the lower fatty acid release observed in SPI matrices, where undigested protein aggregates retain oil droplets or lipid residues after intestinal digestion (Figure 5).

In addition, NE-based gels exhibited fewer lipid residues (stained in red) after in vitro digestion, particularly in WPI samples. Generally, the size of oil droplets in emulsions significantly affects FFA release, as lipase must adsorb onto the oil droplet surface to access the substrate [84]. Therefore, reducing droplet size increases the interfacial area, promoting both the rate and total FFA released over time [67], which agrees with our findings. However, this was not the case for vitamin D bioaccessibility, since the NE-based gel elaborated with WPI showed the lowest bioaccessibility. This fact can be attributed to the degree of proteolysis in the gels. It is well known that WPI is more digestible than plant proteins [85]. As a consequence of this increased proteolysis, WPI generates peptides during digestion that can interact with vitamin D due to their hydrophobic nature, which prevents its solubilization in mixed micelles and, consequently, decreases its bioaccessibility [86]. Therefore, future studies should examine the effect of proteolysis on the bioaccessibility of liposoluble components, such as vitamin D.

## 3. Conclusions

This study demonstrates that hydrocolloid mixture, soy protein isolate (SPI), whey protein isolate (WPI), agar (AG), and κ-carrageenan (CAR), and emulsion droplet size, micrometric (CE) and nanometric (NE) droplet sizes, offer a viable strategy for designing emulsion-based gels with rheological and structural properties aligned with the swallowing needs of older adults. The findings indicate that the physical properties of the gels are affected by hydrocolloid nature and structure, and the intermolecular interactions between proteins and polysaccharides. All gels exhibited pseudoplastic, non-Newtonian behavior, with viscosity values that categorize them as thick foods that do not require mastication, according to the IDDSI guidelines. Given their textural characteristics, these gels are ideal for easy, safe swallowing and are suitable for the swallowing needs of older adults. Regarding in vitro digestion, the percentage of free fatty acids (FFAs) released depends mainly on the protein type and droplet size of the emulsion-based gels. The NE + WPI gels formed aggregates with heterogeneously distributed oil droplets, which promoted the release of FFAs during intestinal digestion. However, in terms of vitamin D bioaccessibility, the lower bioaccessibility observed in WPI gels is presumably related to their higher susceptibility to proteolysis during gastric digestion. These insights provide valuable knowledge for the development of texture-modified foods that support safer swallowing and improved nutrient delivery in aging populations. Therefore, the incorporation of hydrocolloid mixtures (WPI and AG) and emulsions with different droplet sizes enables the development of flan-type desserts tailored to the nutritional and sensory needs of older adults, contributing to improvements in their nutritional status and quality of life.

Despite the relevance of these findings, this study has some limitations, particularly those inherent to in vitro digestion models, which do not fully replicate the physiological complexity of human digestion. Consequently, future research should incorporate in vivo digestion studies that more accurately reflect the gastrointestinal conditions of older adults, in order to validate lipid digestibility, as well as to further elucidate the role of proteolysis in modulating the bioaccessibility of lipophilic nutrients such as vitamin D. Furthermore, sensory evaluation and acceptability assessments will be essential to determine consumer perception and to establish the feasibility of integrating these gels into food products specifically targeted to older populations.

## 4. Materials and Methods

### 4.1. Materials

Emulsions were prepared with canola oil (Belmont, Watts, San Bernardo, Chile) and vitamin D_3_ (cholecalciferol in medium-chain triglyceride oil 1.0 Mio IU/g with α-tocopherol as stabilizer, DSM, Aesch, Switzerland) as lipid phase; soybean lecithin (Metarin P Cargill, Blumos, Santiago, Chile), modified starch (Capsul^®^, Ingredion, Westchester, IL, USA), and food grade polysorbate 80 (P1754, Sigma-Aldrich, St. Louis, MO, USA) as emulsifiers; and purified water obtained through a reverse osmosis system (Vigaflow, Santiago, Chile) as an aqueous phase. Also, whey protein isolate (88% d.b. protein, Provon 292, Blumos, Santiago, Chile), soy protein isolate (90% d.b. protein, Angelini Organics, Santiago, Chile), agar (Tractor Bean, Santiago, Chile), and κ-carrageenan (Sabores y fragancias comercial, Santiago, Chile) were used as gelling agents of emulsions.

For in vitro digestion assays, the following enzymes were used for the preparation of simulated fluids: porcine pepsin (3200 units/ mg protein, P6887), porcine pancreatin (8 × USP specifications, P7545), and bile extract from porcine (B8631) (Sigma-Aldrich, St. Louis, MO, USA). Also, for the preparation of stock solutions of each phase (oral, gastric and intestinal), monopotassium phosphate (KH_2_PO_4_), sodium bicarbonate (NaHCO_3_), potassium chloride (KCl), sodium chloride (NaCl), hexahydrate magnesium chloride (MgCl_2_(H_2_O)_6_), ammonium carbonate ((NH_4_)_2_CO_3_), calcium chloride dihydrate (CaCl_2_(H_2_O)_2_), sodium hydroxide (NaOH) and hydrochloric acid (HCl) were used, and purchased from Merck (Darmstadt, Germany) or Winkler (Santiago, Chile). For confocal microscopy analysis, a Nile Red solution (in polyethylene glycol) (Sigma-Aldrich, St. Louis, MO, USA) was used to stain the samples.

### 4.2. Emulsions and Emulsion-Filled Gels Preparation

The emulsions were prepared following the methodology proposed by Arancibia et al. [87] through three sequential steps: (i) The emulsifiers (8% *w*/*w*), consisting of 0.5% *w*/*w* polysorbate 80, 1% *w*/*w* Capsul^®^, and 6.5% *w*/*w* soybean lecithin, were dispersed in purified water (82% *w*/*w*) using magnetic stirring (AREX, Velp Scientifica, Usmate Velate, Italy) at 500 rpm for 60 min. (ii) Next, a pre-emulsion was prepared by incorporating the lipid phase (10% *w*/*w*), which included 9.5% *w*/*w* canola oil and 0.5% *w*/*w* vitamin D_3_, into the aqueous phase using a high-speed homogenizer (Ultraturrax T25, IKA, Staufen, Germany) operating at 12,000 rpm for 30 min. (iii) Finally, the pre-emulsion was subjected to a high-pressure homogenization process (APV2000, SPX Flow, Bydgoszcz, Poland) to produce the final emulsions. The process parameters for conventional emulsions (CE) were set to 300 bar for 1 cycle, while those for nanoemulsions (NE) were set to 700 bar for 5 cycles.

To prepare emulsion-filled gels, a mixture of hydrocolloids which includes proteins: whey isolate (WPI) and soy protein isolate (SPI), and polysaccharides: agar (AG) and κ-carrageenan (CAR), was incorporated into the emulsions (CE and NE) produced in the previous step. Table 4 presents the nomenclature and formulation of the samples. For gel preparation, the protocol proposed by Riquelme et al. [22] was followed. It is important to note that the hydrocolloid concentrations were selected based on RSM optimization to obtain gels with textures suitable for older adults (i.e., requiring no chewing). First, proteins were dispersed into the emulsions using a magnetic stirrer (AREX, Velp Scientifica, Usmate Velate, Italy) at 600 rpm for 120 min. The resulting dispersions were then refrigerated at 4 ± 1 °C for 24 h to ensure proper protein hydration. Next, the polysaccharides (0.75% *w*/*w* of either AG or CAR) were added to the dispersion. This mixture was heated to 90 °C for 30 min in a thermostatically controlled bath (B04390, Memmert, Schwabach, Germany) while mechanically stirred at 50 rpm (BS, Velp Scientifica, Usmate Velate, Italy). Finally, the resulting sol (pre-gel) was allowed to cool to room temperature (25 °C) in sealed plastic containers and stored at 4 ± 1 °C for 24 h. At least two batches of each sample were prepared for characterization and in vitro digestion assays.

### 4.3. Emulsions Characterization

The droplet size of the conventional emulsion (CE) was determined using an optical microscope (Stemi DV4, Carl Zeiss, Deutschland, Germany) equipped with a digital camera (EOS Rebel T3, Canon, Tokyo, Japan). A 20 μL sample was placed on a slide, and 5 to 6 images were captured at a magnification of 100×. A minimum of 200 drops was analyzed to calculate the average diameter using Motic Images Plus 2.0 software. The droplet size, expressed as the Sauter diameter (D_3,2_), was calculated using Equation (1).

(1) $$D_{3,2}=\frac{n_{i}d_{i}^{3}}{n_{i}d_{i}^{2}}$$

where $d_{i}$ represents the diameter of an oil droplet, and $n_{i}$ is the number of droplets with a diameter $d_{i}$.

For nanoemulsions, the droplet size (hydrodynamic diameter) was determined by dynamic light scattering (DLS) using a Nanosizer (S90, Malvern Panalytical, Malvern, UK) at 25 °C. A refractive index of 1.46 was used for the lipid phase, while a refractive index of 1.33 was used for the continuous phase (water). To prevent turbidity, emulsion samples were diluted to 6% *v*/*v* with purified water. Each droplet size measurement represents the average of three hydrodynamic diameter readings from the same sample.

The physical stability of the emulsions was monitored over a five-week storage period at 5 ± 1 °C, corresponding to the standard refrigeration conditions for this type of food product. For this purpose, the backscattering profiles of the samples were recorded using a Turbiscan device (Classic, Formulaction, Toulouse, France) equipped with an 880 nm laser and two detectors. The first sensor was positioned at 0° relative to the incident light (in front of the source) to measure the intensity of transmitted light. The second sensor, located at 135° from the incident-light direction, measured backscattered light (BS). For that, a 25 mL sample of each emulsion (CE and NE) was placed into cylindrical glass tubes (140 mm high and 15 mm in diameter), which provided a sample height of 60 mm. The samples were scanned weekly, and the data were recorded using Turbisoft Lab 2.2 software. The creaming index (CI) of the samples was calculated according to Equation (2).

(2) $$CI(\%)=(\frac{H_{s}}{H_{t}})\times 100$$

where $H_{s}$ corresponds to the height of the emulsion cream layer, and $H_{t}$ is the total height of the emulsion in the measuring tube.

### 4.4. Rheological and Texture Properties of Emulsion-Filled Gels

The flow behavior of emulsion-filled gels was analyzed using a rotational rheometer (RheoLab QC, Anton Paar, Graz, Austria) with a concentric cylinder geometry (CC27, Anton Paar, Graz, Austria). Before conducting the measurements, samples were loaded into the geometry and allowed to rest for 10 min to stabilize their structure and equilibrate to the test temperature of 37 °C. Flow curves were generated by recording the shear stress values during a linear increase in shear rate from 1 to 100 s^−1^ for 60 s, followed by a reverse sequence over the same duration. The descending flow curves were fitted to the Ostwald-de Waele model (Equation (3)) to determine the flow parameters.

(3) $$\sigma =K\gamma^{n}$$

where σ is shear stress (Pa), the shear rate (s^−1^), K is the consistency index (Pa·s), and n is the flow behavior index. Additionally, the apparent viscosity at a shear rate of 50 s^−1^ was calculated, as this is considered the shear rate during the swallowing of semisolid foods [88].

The textural properties of the emulsion-filled gels were evaluated using Texture Profile Analysis (TPA) conducted on a texture analyzer (Z0.5, Zwick, Ulm, Germany) equipped with a cylindrical aluminum probe (5 cm diameter). The samples were compressed perpendicularly to 20% of their initial height for two consecutive cycles at a constant speed of 0.1 mm/s under room temperature conditions (25 ± 1 °C). All gels maintained a cylindrical shape with a diameter of 3.6 cm and a height of 1.25 cm before compression. Finally, force-time deformation curves were recorded, and parameters such as hardness, adhesiveness, elasticity, gumminess, and cohesiveness were calculated using TestXpert II software (Zwick, Ulm, Germany).

### 4.5. In Vitro Digestion of Emulsion-Filled Gels

#### 4.5.1. In Vitro Digestion

Emulsion-filled gels were subjected to in vitro lipid digestion using a static gastrointestinal model based on the INFOGEST protocol, with some modifications. This protocol included simulating the oral, gastric, and intestinal phases of digestion. Additionally, the protocol was adapted to accommodate specific conditions for older populations [89]. The specifications were as follows:

- Oral Phase: A 25 g sample was chewed by a male volunteer (aged over 73 years old), following a structured protocol for bolus formation and collection: (1) The volunteer abstained from food for at least two hours before the experiment. (2) The volunteer rinsed their mouth with water before starting the protocol. (3) The gel sample was divided into three portions to facilitate oral handling. Each portion was placed in the oral cavity and held for 10 s without chewing or swallowing. (4) The formed bolus was collected in a 50 mL centrifuge tube until all portions had been processed. To complete the bolus preparation, 20 mL of preheated (37 °C) simulated oral fluid (SOF) and 125 µL of 0.3 M CaCl_2_ were added, maintaining a 1:1 (*v*/*v*) ratio of bolus to SOF, as specified by the INFOGEST protocol. Ethical approval for the involvement of human subjects in this study was granted by the Ethics Committee of the Universidad de Santiago de Chile (reference number 228, dated 19 April 2023).
- Gastric Phase: The bolus obtained in the previous phase (50 mL) was mixed with simulated gastric fluid (SGF) at a 1:1 ratio. The SGF consisted of 40 mL of gastric electrolyte solution, 25 µL of 0.3 M CaCl_2_, and 2.5 mL of pepsin solution (1500 U/mL in the final mixture). The mixture was incubated in a temperature-controlled bath at 37 °C with constant agitation at 200 rpm for 180 min. The pH of the sample was adjusted using 0.5 M HCl with an automatic titration device (902 Titrando, Metrohm, Riverview, FL, USA) in three stages: (a) to pH 4 at a rate of 0.15 mL/min for 20 min, (b) to pH 3 at a rate of 0.08 mL/min for 40 min, and (c) to pH 2 at a rate of 0.07 mL/min for 120 min.
- Intestinal phase: The chyme was mixed with simulated intestinal fluid (SIF) in a 1:1 ratio. The SIF consisted of 42.5 mL of intestinal electrolyte solution, 200 µL of 0.3 M CaCl_2_, 12.5 mL of a bile solution (10 mmol/L), and 25 mL of pancreatin solution (80 U/mL in the final mixture). This mixture was incubated at 37 °C for 120 min with constant agitation at 200 rpm. The pH was maintained at 7 using an automatic titration device, which added 0.5 M NaOH to neutralize the free fatty acids (FFAs) released during lipolysis during digestion.

#### 4.5.2. Quantification of Lipid Digestion

The release of free fatty acids (FFAs) during in vitro intestinal digestion was quantified using Equation (4) [90].

(4) $$FFA(\%)=\frac{V_{NaOH(t)\;}\times M_{NaOH}\times {MW}_{oil}}{m_{oil}\times 2}$$

where $V_{NaOH(t)}$ represents the volume (L) of the NaOH solution used to neutralize the FFAs generated during digestion, $M_{NaOH}$ is the molar concentration (mol/L) of NaOH solution used for titration of the sample, ${MW}_{oil}$ is the molecular weight of the lipid phase (g/mol), and $m_{oil}$ is the initial lipid mass (g). To compare the degree of digestion of the evaluated samples, the final degree of digestion was expressed as the total percentage of FFAs released by the end of the intestinal phase.

#### 4.5.3. Vitamin D Bioaccessibility

The bioaccessibility of vitamin D_3_ was assessed following the in vitro digestion process, as described by Schoener et al. [91]. First, mixed micelles were extracted from the digested samples. For that, a 25 mL aliquot of the intestinal phase (chyle) mixture was centrifuged in a Universal 32R centrifuge (Hettich, Salford, UK) at 9000 rpm and 4 °C for 60 min. This centrifugation process separated the samples into two distinct phases: a sediment phase at the bottom of the tube and a clear phase on top, containing the mixed micelles that included the solubilized bioactive compounds.

Subsequently, vitamin D_3_ extraction was performed by mixing 2 mL of the chyle (digest sample) or the mixed micelles with 4 mL of a hexane: ethanol (1:1) solution. This mixture was vortexed for 30 s using a Vortex (FinePCR, Gyeonggi-do, Korea). The mixture was then centrifuged again at 2000 rpm for 2 min to collect the organic phase (supernatant). The solvent was removed using an atmospheric nitrogen system. To the dry samples, 1 mL of methanol was added, and the mixture was filtered through a PTFE syringe filter with a 0.45 μm pore size.

The quantification of vitamin D_3_ was performed using a high-resolution liquid chromatograph (Alliance e2695, Waters, Queenstown, Singapore) fitted with a diode array detector (DAD) (2998 PDA detector, Waters, Queenstown, Singapore). The measurement conditions were as follows: a C18 column (4.6 mm diameter × 250 mm length, 0.5 μm thickness, Waters, Queenstown, Singapore) was used as the stationary phase at 25 °C; the mobile phase consisted of methanol and water (95:5); the flow rate was set to 0.8 mL/min; the injection volume was 20 μL; and detection was carried out at a wavelength of 265 nm. The obtained data were analyzed using Empower 3 software (Waters, Queenstown, Singapore). Finally, the bioaccessibility of vitamin D_3_ was calculated using Equation (5) based on its quantification.

(5) $$Bioaccessibility\;(\%)=\frac{C_{micelles}}{C_{chyle}}\times 100$$

where $C_{micelles}$ and $C_{chyle}$ are the concentrations of vitamin D_3_ determined in the micelles mixed and chyle, respectively.

#### 4.5.4. Microstructure

The microstructure of emulsion-filled gels was examined before and after digestion using confocal laser scanning microscopy (CLSM). To prepare the samples, 200 μL of each sample obtained before and after in vitro digestion was stained with 100 μL of a Nile Red solution (0.1 g/L in polyethylene glycol). Next, 150 μL of each stained sample was placed between two coverslips in the observation chamber using a 40× oil-immersion objective lens. The excitation and emission wavelengths for the Nile Red dye were set at 534 nm and 560 nm, respectively. The resulting images were analyzed using ZEN Lite 2.3 software (Carl Zeiss, Deutschland, Germany).

### 4.6. Statistical Analysis

The instrumental data from at least 4 measurements were analyzed using the XLSTAT program (Lumivero, Denver, CO, USA). An analysis of variance (ANOVA) was performed with three factors: type of protein and polysaccharide, and droplet size. A post hoc Fisher’s Least Significant Difference (LSD) test was conducted at α = 0.05 to identify significant differences among the samples for the various parameters studied.

## Figures and Tables

**Table 1:** Physical characterization and stability of the emulsions with different droplet sizes.

Emulsion Type	Droplet Size	Polydispersity Index [-]	Creaming Index [%]
Conventional emulsion (CE)	20.7 ± 0.4 ^b^ µm	-	9.4 ± 0.2 ^a^
Nanoemulsion (NE)	193.5 ± 2.6 ^a^ nm	0.13 ± 0.04	3.8 ± 0.2 ^b^

Note: Each value represents the mean ± SD. Different letters indicate significant differences (Fisher; *p* < 0.05) in the parameters (same column) for different samples.

**Table 2:** Flow parameters of emulsion-filled gels with different droplet sizes and hydrocolloid type.

Sample	*K* [Pa s]	*n*	η_50s−1_ [Pa s]
CE + WPI + CAR	27.39 ± 1.37 ^b^	0.34 ± 0.01 ^a^	2021 ± 96 ^b^
CE + WPI + AG	84.12 ± 1.53 ^c^	0.25 ± 0.01 ^c^	5494 ± 107 ^c^
CE + SPI + CAR	18.95 ± 1.63 ^a^	0.23 ± 0.01 ^d^	924 ± 115 ^a^
CE + SPI + AG	26.16 ± 1.44 ^b^	0.31 ± 0.01 ^b^	2121 ± 101 ^b^
NE + WPI + CAR	28.63 ± 1.77 ^b^	0.35 ± 0.01 ^a^	2158 ± 136 ^b^
NE + WPI + AG	89.67 ± 1.37 ^d^	0.25 ± 0.01 ^c^	5903 ± 96 ^d^
NE + SPI + CAR	18.73 ± 2.16 ^a^	0.21 ± 0.01 ^d^	925 ± 152 ^a^
NE + SPI + AG	29.34 ± 1.37 ^b^	0.31 ± 0.01 ^b^	2262 ± 152 ^b^

Note: CE: conventional emulsion, NE: nanoemulsion, SPI: soy protein isolate, WPI: whey protein isolate, AG: agar, and CAR: κ-carrageenan. *K*: consistency index, *n*: flow index, and η_50__s__−1_: apparent viscosity a 50 s^−1^. Each value represents the mean ± SD. Different letters indicate significant differences (Fisher; *p* < 0.05) in the parameters (same column) for different samples.

**Table 3:** Texture parameters of emulsion-filled gels with different droplet sizes and hydrocolloid type.

Sample	Hardness [N/m^2^]	Adhesiveness [J/m^2^]	Cohesiveness [-]
CE + WPI + CAR	495 ± 38 ^cd^	0.05 ± 0.01 ^bc^	0.57 ± 0.02 ^bc^
CE + WPI + AG	682 ± 46 ^e^	0.06 ± 0.01 ^c^	0.63 ± 0.02 ^d^
CE + SPI + CAR	297 ± 38 ^a^	0.02 ± 0.01 ^a^	0.43 ± 0.02 ^a^
CE + SPI + AG	412 ± 38 ^bc^	0.03 ± 0.01 ^ab^	0.56 ± 0.02 ^b^
NE + WPI + CAR	509 ± 38 ^cd^	0.05 ± 0.01 ^c^	0.57 ± 0.02 ^bc^
NE + WPI + AG	586 ± 39 ^de^	0.06 ± 0.01 ^c^	0.62 ± 0.02 ^cd^
NE + SPI + CAR	339 ± 48 ^a^	0.03 ± 0.01 ^a^	0.46 ± 0.02 ^a^
NE + SPI + AG	353 ± 38 ^a^	0.04 ± 0.01 ^abc^	0.53 ± 0.02 ^b^

Note: CE: conventional emulsion, NE: nanoemulsion, SPI: soy protein isolate, WPI: whey protein isolate, AG: agar, and CAR: κ-carrageenan. Each value represents the average ± SD. Different letters indicate significant differences (Fisher; *p* < 0.05) in the parameters (same column) for different samples.

**Table 4:** Nomenclature and formulation of emulsion-filled gels with different droplet sizes and hydrocolloid type.

Sample	Emulsion Type	Type and Concentration of Hydrocolloid	
		Protein (% *w*/*w*)	Polysaccharide (% *w*/*w*)
CE + WPI + CAR	Conventional emulsion (CE)	Whey protein isolate (WPI) 5.97%	*κ*-carrageenan (CAR) 0.75%
CE + WPI + AG			Agar (AG) 0.75%
CE + SPI + CAR		Soy protein isolate (SPI) 5.83%	*κ*-carrageenan (CAR) 0.75%
CE + SPI + AG			Agar (AG) 0.75%
NE + WPI + CAR	Nanoemulsion (NE)	Whey protein isolate (WPI) 5.97%	*κ*-carrageenan (CAR) 0.75%
NE + WPI + AG			Agar (AG) 0.75%
NE + SPI + CAR		Soy protein isolate (SPI) 5.83%	*κ*-carrageenan (CAR) 0.75%
NE + SPI + AG			Agar (AG) 0.75%

Note: CE: conventional emulsion, NE: nanoemulsion, SPI: soy protein isolate, WPI: whey protein isolate, AG: agar, and CAR: κ-carrageenan. The total protein concentration in gels was 5.25% *w*/*w* and was calculated through the dry base concentration of whey protein isolate (88%) and soy protein isolate (90%), depending on the gel type.

## Data Availability

Dataset available on request from the authors.

## References

[1] WHO Ageing and Health. https://www.who.int/news-room/fact-sheets/detail/ageing-and-health.

[2] Cava E., Lombardo M. (2024). Nutritional strategies for ageing populations: Focusing on dysphagia and geriatric nutritional needs. Eur. J. Clin. Nutr..

[3] Hernández-Olivas E., Muñoz-Pina S., Andrés A., Heredia A. (2020). Impact of elderly gastrointestinal alterations on in vitro digestion of salmon, sardine, sea bass and hake: Proteolysis, lipolysis and bioaccessibility of calcium and vitamins. Food Chem..

[4] Neri M.C., d’Alba L. (2021). Nutrition and healthy aging: Prevention and treatment of gastrointestinal diseases. Nutrients.

[5] Fernandes J.M., Araújo J.F., Vieira J.M., Pinheiro A.C., Vicente A.A. (2021). Tackling older adults’ malnutrition through the development of tailored food products. Trends Food Sci. Technol..

[6] Bari A.B.A., Samuel P.J., Pathak S., Banerjee A., Duttaroy A.K. (2023). Macronutrients and their roles in aging. Evidence-Based Functional Foods for Prevention of Age-Related Diseases.

[7] Bojang K.P., Manchana V. (2023). Nutrition and healthy aging: A review. Curr. Nutr. Rep..

[8] Fekete M., Szarvas Z., Fazekas-Pongor V., Feher A., Csipo T., Forrai J., Dosa N., Peterfi A., Lehoczki A., Tarantini S. (2022). Nutrition strategies promoting healthy aging: From improvement of cardiovascular and brain health to prevention of age-associated diseases. Nutrients.

[9] Mavar M., Sorić T., Bagarić E., Sarić A., Matek Sarić M. (2024). The power of vitamin D: Is the future in precision nutrition through personalized supplementation plans?. Nutrients.

[10] Kupisz-Urbańska M., Płudowski P., Marcinowska-Suchowierska E. (2021). Vitamin D deficiency in older patients—Problems of sarcopenia, drug interactions, management in deficiency. Nutrients.

[11] Yiu C.C.Y., Liang S.W., Mukhtar K., Kim W., Wang Y., Selomulya C. (2023). Food emulsion gels from plant-based ingredients: Formulation, processing, and potential applications. Gels.

[12] Xu W., Li X., Wen X., Wang Y., Sun B., Xu D. (2025). Calcium ion-regulated oil body-filled pea protein isolate–inulin emulsion gels for dysphagia-oriented products. Food Hydrocoll..

[13] Boonlao N., Ruktanonchai U.R., Anal A.K. (2022). Enhancing bioaccessibility and bioavailability of carotenoids using emulsion-based delivery systems. Colloids Surf. B Biointerfaces.

[14] Li R., Guo Y., Dong A., Yang X. (2023). Protein-based emulsion gels as materials for delivery of bioactive substances: Formation, structures, applications and challenges. Food Hydrocoll..

[15] Fontes-Candia C., Martínez J.C., López-Rubio A., Salvia-Trujillo L., Martín-Belloso O., Martínez-Sanz M. (2022). Emulsion gels and oil-filled aerogels as curcumin carriers: Nanostructural characterization of gastrointestinal digestion products. Food Chem..

[16] Yang J., Wan L., Duan X., Wang H., Yang Z., Liu F., Xu X., Pan S. (2022). Potential low-calorie model that inhibits free fatty acid release and helps curcumin deliver in vitro: Ca^2+^-induced emulsion gels from low methyl-esterified pectin with erythritol. Int. J. Biol. Macromol..

[17] Li S., Li P., Wang J., Lu Y., Chen Y., Zhao Z., Jiang J., Cheng X., Bi L. (2025). Investigation of the stability and bioaccessibility of β-carotene encapsulated in emulsion gels with nonspherical droplets. ACS Food Sci. Technol..

[18] Zhao K., Hao Y., Gan J., Ye H., Shen X. (2025). Development of quinoa protein emulsion gels to deliver curcumin: Influence of oil type. J. Food Eng..

[19] Abdullah, Liu L., Javed H.U., Xiao J. (2022). Engineering emulsion gels as functional colloids emphasizing food applications: A review. Front. Nutr..

[20] Wang Z., Deng Y., Zhang Y., Wei Z., Wan Z., Li C., Tang X., Zhao Z., Zhou P., Li P. (2023). Impacts of citric acid concentration and pH on rheological properties of cold-set whey protein fibril hydrogels. LWT.

[21] Wang Z., Deng Y., Zhang Y., Tang X., Zhou P., Li P., Zhao Z., Wang Z., Liu G., Zhang M. (2024). Fibrous whey protein–mediated homogeneous and soft-textured emulsion gels for elderly: Enhancement of curcumin bioaccessibility. Food Chem..

[22] Riquelme N., Savignones C., López A., Zúñiga R.N., Arancibia C. (2023). Effect of gelling agent type on the physical properties of nanoemulsion-based gels. Colloids Interfaces.

[23] Shen J., Chen Y., Li X., Zhou X., Ding Y. (2024). Enhanced probiotic viability in innovative double-network emulsion gels: Synergistic effects of whey protein concentrate–xanthan gum complex and κ-carrageenan. Int. J. Biol. Macromol..

[24] Fu J., Zhan Z., Duan Q., Yang Y., Xie H., Dong X., Zhang H., Yu L. (2025). Application of soybean protein isolate–polysaccharide hybrid emulsion gels as alternative fats in plant-based meats. LWT.

[25] Li R., Wang N., Ma C., Wang J., Yang X. (2025). Construction and formation mechanism of phase-change polysaccharide–protein composite emulsion gels: For simultaneous printing of complex food structures. Food Hydrocoll..

[26] Yiasmin M.N., Al Azad S., Easdani M., Islam M.S., Hussain M., Cao W., Chen N., Uriho A., Asaduzzaman M., Liu C. (2025). The state-of-the-art on exploring polysaccharide-protein interactions and its mechanisms, stability, and their role in food systems. Food Rev. Int..

[27] Campbell W.W., Deutz N.E., Volpi E., Apovian C.M. (2023). Nutritional interventions: Dietary protein needs and influences on skeletal muscle of older adults. J. Gerontol. A Biol. Sci. Med. Sci..

[28] Yang X., Li A., Li D., Guo Y., Sun L. (2021). Applications of mixed polysaccharide–protein systems in fabricating multi-structured binary food gels: A review. Trends Food Sci. Technol..

[29] Meng Y., Liang Z., Zhang C., Hao S., Han H., Du P., Li A., Shao H., Li C., Liu L. (2021). Ultrasonic modification of whey protein isolate: Implications for the structural and functional properties. LWT.

[30] Zheng L., Regenstein J.M., Zhou L., Wang Z. (2022). Soy protein isolates: A review of their composition, aggregation, and gelation. Compr. Rev. Food Sci. Food Saf..

[31] Pascuta M.S., Varvara R.A., Teleky B.E., Szabo K., Plamada D., Nemeş S.A., Mitrea L., Martău G.A., Ciont C., Călinoiu L.F. (2022). Polysaccharide-based edible gels as functional ingredients: Characterization, applicability, and human health benefits. Gels.

[32] Kumar A., Hart P., Thakur V.K. (2024). Seaweed based hydrogels: Extraction, gelling characteristics, and applications in the agriculture sector. ACS Resour. Manag..

[33] Mushtaq A., Wani S.M., Malik A.R., Gull A., Ramniwas S., Nayik G.A., Ercisli S., Marc R.A., Ullah R., Bari A. (2023). Recent insights into nanoemulsions: Their preparation, properties and applications. Food Chem. X.

[34] Carpenter J., Pinjari D.V., Kumar Saharan V., Pandit A.B. (2022). Critical review on hydrodynamic cavitation as an intensifying homogenizing technique for oil-in-water emulsification: Theoretical insight, current status, and future perspectives. Ind. Eng. Chem. Res..

[35] Di Giorgio L., Salgado P.R., Mauri A.N. (2022). Fish oil-in-water emulsions stabilized by soy proteins and cellulose nanocrystals. Carbohydr. Polym. Technol. Appl..

[36] McClements D.J., Jafari S.M. (2018). General aspects of nanoemulsions and their formulation. Nanoemulsions.

[37] Kupikowska-Stobba B., Domagała J., Kasprzak M.M. (2024). Critical review of techniques for food emulsion characterization. Appl. Sci..

[38] Huang J., Chen X., Su D., Chen L., Chen C., Jin B. (2023). Molecular mechanisms affecting the stability of high internal phase emulsions of zein–soy isoflavone complexes fabricated with ultrasound-assisted dynamic high-pressure microfluidization. Food Res. Int..

[39] Farjami T., Madadlou A. (2019). An overview on preparation of emulsion-filled gels and emulsion particulate gels. Trends Food Sci. Technol..

[40] McClements D.J., Rao J. (2011). Food-grade nanoemulsions: Formulation, fabrication, properties, performance, biological fate, and potential toxicity. Crit. Rev. Food Sci. Nutr..

[41] Babu A., Shams R., Dash K.K., Shaikh A.M., Kovács B. (2024). Protein–polysaccharide complexes and conjugates: Structural modifications and interactions under diverse treatments. J. Agric. Food Res..

[42] Methacanon P., Gamonpilas C., Kongjaroen A., Buathongjan C. (2021). Food polysaccharides and roles of rheology and tribology in rational design of thickened liquids for oropharyngeal dysphagia: A review. Compr. Rev. Food Sci. Food Saf..

[43] Martínez-Padilla L.P. (2024). Rheology of liquid foods under shear flow conditions: Recently used models. J. Texture Stud..

[44] Mirazimi F., Saldo J., Sepulcre F., Gràcia A., Pujola M. (2022). Enriched potato puree with soy protein for dysphagia patients using 3D printing. Food Front..

[45] Wagner J., Biliaderis C.G., Moschakis T. (2020). Whey proteins: Musings on denaturation, aggregate formation and gelation. Crit. Rev. Food Sci. Nutr..

[46] Martínez-Sanz M., Ström A., Lopez-Sanchez P., Knutsen S.H., Ballance S., Zobel H.K., Sokolova A., Gilbert E.P., López-Rubio A. (2020). Advanced structural characterization of agar-based hydrogels: Rheological and small-angle scattering studies. Carbohydr. Polym..

[47] Ding R., Zhang M., Zhu Q., Qu Y., Jia X., Yin L. (2023). Curcumin-loaded zein–alginate nanogels with “core–shell” structure: Formation, characterization and simulated digestion. Int. J. Biol. Macromol..

[48] Elmarhoum S., Ako K., Munialo C.D., Rharbi Y. (2023). Helicity degree of carrageenan conformation determines the polysaccharide and water interactions. Carbohydr. Polym..

[49] Qiao D., Zhang Y., Lin L., Li K., Zhu F., Wang G., Xi G., Jiang F., Zhang B., Xie F. (2024). Revealing the role of λ-carrageenan on the enhancement of gel-related properties of acid-induced soy protein isolate/λ-carrageenan system. Food Hydrocoll..

[50] Kierulf A.V., Whaley J.K., Liu W., Smoot J.T., Jenab E., Perez Herrera M., Abbaspourrad A. (2022). Heat- and shear-reversible networks in food: A review. Compr. Rev. Food Sci. Food Saf..

[51] Ibañez F.C., Gómez I., Merino G., Beriain M. (2019). Textural characteristics of safe dishes for dysphagic patients: A multivariate analysis approach. Int. J. Food Prop..

[52] Peña-Chávez R.E., Schaen-Heacock N.E., Hitchcock M.E., Kurosu A., Suzuki R., Hartel R.W., Ciucci M.R., Rogus-Pulia N.M. (2023). Effects of food and liquid properties on swallowing physiology and function in adults. Dysphagia.

[53] IDDSI The IDDSI Framework: International Dysphagia Diet Standardization Initiative. https://www.iddsi.org/images/Publications-Resources/DetailedDefnTestMethods/English/V2DetailedDefnEnglish31july2019.pdf.

[54] Hadde E.K., Chen J. (2021). Texture and texture assessment of thickened fluids and texture-modified food for dysphagia management. J. Texture Stud..

[55] Rahman M.S., Al-Attabi Z.H., Al-Habsi N., Al-Khusaibi M., Khan M.S., Shafiur Rahman M. (2021). Measurement of instrumental texture profile analysis (TPA) of foods. Techniques to Measure Food Safety and Quality.

[56] Lin D., Kelly A.L., Miao S. (2021). The role of mixing sequence in structuring O/W emulsions and emulsion gels produced by electrostatic protein–polysaccharide interactions between soy protein isolate-coated droplets and alginate molecules. Food Hydrocoll..

[57] Ibañez F.C., Merino G., Marín-Arroyo M.R., Beriain M.J. (2022). Instrumental and sensory techniques to characterize the texture of foods suitable for dysphagic people: A systematic review. Compr. Rev. Food Sci. Food Saf..

[58] Wada S., Kawate N., Mizuma M. (2017). What type of food can older adults masticate: Evaluation of mastication performance using color-changeable chewing gum. Dysphagia.

[59] Cichero J.A.Y. (2020). Evaluating chewing function: Expanding the dysphagia field using food oral processing and the IDDSI framework. J. Texture Stud..

[60] Nishinari K., Fang Y., Rosenthal A.J. (2019). Human oral processing and texture profile analysis parameters: Bridging the gap between sensory evaluation and instrumental measurements. J. Texture Stud..

[61] Munialo C.D., Kontogiorgos V., Euston S.R., Nyambayo I. (2020). Rheological, tribological and sensory attributes of texture-modified foods for dysphagia patients and the elderly: A review. Int. J. Food Sci. Technol..

[62] Makame J., Nolden A.A., Emmambux M.N. (2023). Texture properties of foods targeted for individuals with limited oral processing capabilities: The elderly, dysphagia, and head and neck cancer patients. Food Funct..

[63] Li S., Li P., Wang J., Lu Y., Chen Y., Zhao Z., Bi L. (2024). Characterization and stability of low-oil emulsion gels with newly shaped droplets stabilized by camellia saponin and κ-carrageenan. Food Hydrocoll..

[64] Riquelme N., Robert P., Troncoso E., Arancibia C. (2020). Influence of particle size and hydrocolloid type on lipid digestion of thickened emulsions. Food Funct..

[65] Xiao J., Tian W., Abdullah, Wang H., Chen M., Huang Q., Zhang M., Lu M., Song M., Cao Y. (2024). Updated design strategies for oral delivery systems: Maximized bioefficacy of dietary bioactive compounds achieved by inducing proper digestive fate and sensory attributes. Crit. Rev. Food Sci. Nutr..

[66] Guo Y., Yang X., Bao Y., Zhao X., Huang L., Chen Z., Ma Y., Lu W. (2022). Investigation of the in vitro digestion fate and oxidation of protein-based oleogels prepared by pine nut oil. LWT.

[67] McClements D.J., Li Y. (2010). Structured emulsion-based delivery systems: Controlling the digestion and release of lipophilic food components. Adv. Colloid Interface Sci..

[68] Infantes-Garcia M.R., Verkempinck S.H.E., Gonzalez-Fuentes P.G., Hendrickx M.E., Grauwet T. (2021). Lipolysis product formation during in vitro gastric digestion is affected by the emulsion interfacial composition. Food Hydrocoll..

[69] Mackie A., Mulet-Cabero A.I., Torcello-Gómez A. (2020). Simulating human digestion: Developing our knowledge to create healthier and more sustainable foods. Food Funct..

[70] Günter E.A., Martynov V.V., Belozerov V.S., Martinson E.A., Litvinets S.G. (2020). Characterization and swelling properties of composite gel microparticles based on pectin and κ-carrageenan. Int. J. Biol. Macromol..

[71] Qazi H.J., Ye A., Acevedo-Fani A., Singh H. (2021). In vitro digestion of curcumin–nanoemulsion-enriched dairy protein matrices: Impact of the type of gel structure on the bioaccessibility of curcumin. Food Hydrocoll..

[72] Das T., Chatterjee N., Chakraborty A., Banerjee A., Haiti S.B., Datta S., Chattopadhyay H., Dhar P. (2023). Fabrication of rice bran oil nanoemulsion and conventional emulsion with mustard protein isolate as a novel excipient: Focus on shelf-life stability, lipid digestibility and cellular bioavailability. Food Hydrocoll. Health.

[73] Gonzalez C.B., Simpson R., Vega-Castro O., Del Campo V., Pinto M., Fuentes L., Nuñez H., Young A.K., Ramírez C. (2020). Effect of particle size on in vitro intestinal digestion of emulsion-filled gels: Mathematical analysis based on the Gallagher–Corrigan model. Food Bioprod. Process..

[74] Zhang Y., Han M., Guo Q. (2024). Understanding formation, gastrointestinal breakdown, and application of whey protein emulsion gels: Insights from intermolecular interactions. Compr. Rev. Food Sci. Food Saf..

[75] Guo Y., Liu C., Ma Y., Shen L., Gong Q., Hu Z., Wang Z., Liu X., Guo Z., Zhou L. (2023). Study on the structure, function, and interface characteristics of soybean protein isolate by industrial phosphorylation. Foods.

[76] Liu F., Zhang S., Chen K., Zhang Y. (2023). Fabrication, in vitro digestion and pH-responsive release behavior of soy protein isolate glycation conjugate-based hydrogels. Food Res. Int..

[77] Chen X., Chen Y., Liu Y., Zou L., McClements D.J., Liu W. (2022). Recent progress in improving the bioavailability of nutraceutical-loaded emulsions after oral intake: A review. Compr. Rev. Food Sci. Food Saf..

[78] Guo Y., Xu Y., Zhang T., Wang Y., Liu R., Chang M., Wang X. (2022). Medium- and long-chain structured triacylglycerol enhances vitamin D bioavailability in an emulsion-based delivery system: Combination of in vitro and in vivo studies. Food Funct..

[79] Iddir M., Porras Yaruro J.F., Cocco E., Hardy E.M., Appenzeller B.M., Guignard C., Larondelle Y., Bohn T. (2021). Impact of protein-enriched plant food items on the bioaccessibility and cellular uptake of carotenoids. Antioxidants.

[80] Iddir M., Yaruro J.F.P., Larondelle Y., Bohn T. (2021). Gastric lipase can significantly increase lipolysis and carotenoid bioaccessibility from plant food matrices in the harmonized INFOGEST static in vitro digestion model. Food Funct..

[81] Gómez-Mascaraque L.G., De Pinho S.C. (2021). Microstructural analysis of whey/soy protein isolate mixed gels using confocal Raman microscopy. Foods.

[82] Wang S., Liu X., Zhao G., Li Y., Yang L., Zhu L., Liu H. (2022). Protease-induced soy protein isolate characteristics and structure evolution at the oil–water interface of emulsion. J. Food Eng..

[83] Lu Y., Zhang Y., Yuan F., Gao Y., Mao L. (2021). Emulsion gels with different proteins at the interface: Structures and delivery functionality. Food Hydrocoll..

[84] Acevedo-Fani A., Singh H. (2022). Biophysical insights into modulating lipid digestion in food emulsions. Prog. Lipid Res..

[85] Bailey H., Stein H. (2020). Differences in amino acid digestibility and protein quality among various protein isolates and concentrates derived from cereal grains, plant and dairy proteins. Curr. Dev. Nutr..

[86] Iddir M., Vahid F., Merten D., Larondelle Y., Bohn T. (2022). Influence of proteins on the absorption of lipophilic vitamins, carotenoids and curcumin: A review. Mol. Nutr. Food Res..

[87] Arancibia C., Paredes-Toledo J., Riquelme N. (2024). Oral structural breakdown and sensory perception of plant-based emulsions. Food Hydrocoll..

[88] Gamonpilas C., Kongjaroen A., Methacanon P. (2023). The importance of shear and extensional rheology and tribology as design tools for developing food thickeners for dysphagia management. Food Hydrocoll..

[89] Menard O., Lesmes U., Shani-Levi C.S., Calahorra A.A., Lavoisier A., Morzel M., Rieder A., Feron G., Nebbia S., Mashiah L. (2023). Static in vitro digestion model adapted to the general older adult population: An INFOGEST international consensus. Food Funct..

[90] Li Y., McClements D.J. (2010). New mathematical model for interpreting pH-stat digestion profiles: Impact of lipid droplet characteristics on in vitro digestibility. J. Agric. Food Chem..

[91] Schoener A.L., Zhang R., Lv S., Weiss J., McClements D.J. (2019). Fabrication of plant-based vitamin D_3_-fortified nanoemulsions: Influence of carrier oil type on vitamin bioaccessibility. Food Funct..

